# Targeting cancer stem cells with p53 modulators

**DOI:** 10.18632/oncotarget.8650

**Published:** 2016-04-08

**Authors:** Zhan Zhang, Ling Liu, Roberto Gomez-Casal, Xinhui Wang, Ryo Hayashi, Ettore Appella, Levy Kopelovich, Albert B. DeLeo

**Affiliations:** ^1^ Department of Obstetrics and Gynecology, The Third Affiliated Hospital of Zhengzhou University, Zhengzhou, Henan, China; ^2^ Department of Surgery, Division of Surgical Oncology, Massachusetts General Hospital, Harvard Medical School, Boston, MA, USA; ^3^ University of Pittsburgh Cancer Institute, Pittsburgh, PA, USA; ^4^ Department of Pathology, University of Pittsburgh, Pittsburgh, PA, USA; ^5^ National Cancer Institute, Bethesda, MD, USA; ^6^ Department of Medicine, Weill Cornell Medical College, New York, NY, USA

**Keywords:** CP-31398, PRIMA-1, cancer stem cells, P53 vaccine, T cells

## Abstract

Cancer stem cells (CSC) typically over-express aldehyde dehydrogenase (ALDH). Thus, ALDH^bright^ tumor cells represent targets for developing novel cancer prevention/treatment interventions. Loss of p53 function is a common genetic event during cancer development wherein small molecular weight compounds (SMWC) that restore p53 function and reverse tumor growth have been identified. Here, we focused on two widely studied p53 SMWC, CP-31398 and PRIMA-1, to target ALDH^bright^ CSC in human breast, endometrial and pancreas carcinoma cell lines expressing mutant or wild type (WT) p53. CP-31398 and PRIMA-1 significantly reduced CSC content and sphere formation by these cell lines *in vitro*. In addition, these agents were more effective *in vitro* against CSC compared to cisplatin and gemcitabine, two often-used chemotherapeutic agents. We also tested a combinatorial treatment in methylcholantrene (MCA)-treated mice consisting of p53 SMWC and p53-based vaccines. Yet using survival end-point analysis, no increased efficacy in the presence of either p53 SMWC alone or with vaccine compared to vaccine alone was observed. These results may be due, in part, to the presence of immune cells, such as activated lymphocytes expressing WT p53 at levels comparable to some tumor cells, wherein further increase of p53 expression by p53 SMWC may alter survival of these immune cells and negatively impact an effective immune response. Continuous exposure of mice to MCA may have also interfered with the action of these p53 SMWC, including potential direct interaction with MCA. Nonetheless, the effect of p53 SMWC on CSC and cancer treatment remains of great interest.

## INTRODUCTION

The cancer stem cell (CSC) concept posits the existence of a subpopulation of tumor cells that display “stem cell-like” properties, namely, self-renewal and high tumorigenic potential, including resistance to most currently employed chemo- and radiotherapies [1–3]. Thus, CSC may be largely responsible for the failure of most conventional therapies to control cancer growth and metastases. As such, CSC represents a potential target for the development of novel and effective strategies for cancer prevention/treatment [4, 5].

Although the consensus is that no single marker adequately defines CSC, a considerable amount of data indicates that tumor cells which express elevated levels of aldehyde dehydrogenase (ALDH) activity, ALDH^positive^ cells, have been shown to have CSC-like properties, in particular, the ability to initiate tumor growth at low numbers in immunodeficient mice [6–12]. ALDH is a retinal dehydrogenase implicated in the biosynthesis of retinoic acid, as well as the metabolism of many types of genotoxic agents, including currently administered chemotherapeutic agents [13–15]. Thus, ALDH levels may largely determine chemo- and radio-resistance of CSC [16, 17]. Of particular importance in defining ALDH activity of CSC is the role of a specific ALDH isoform, ALDH1A1 [6]. We previously demonstrated that ALDH1A1 is a human tumor antigen and that ALDH^positive^ cells can be targeted for eradication by ALDH1A1 - specific cytotoxic T cells [18, 19]. Accordingly, we use ALDH as a surrogate marker to identify CSC in human and murine tumor cells. Specifically, the research focuses on tumor cells having 2X the mean fluorescence (MFI) of ALDH^positive^ cells in a tumor, a subpopulation shown to consist of > 90% ALDH^positive^ cells [19].

Targeting aberrantly regulated stem cell signaling pathways such as Hedgehog, Notch and Wingless pathways with small molecular weight compounds (SMWC) is being actively investigated. In this context, loss of function of p53 by direct alteration of *TP53* and/or genes involved in its regulation, is one of the most common genetic events leading up to neoplasia, and is important for developing therapies related to targeting CSC [20, 21]. A number of p53 modulators, consisting of synthetic peptides, organic compounds, and natural products, have been identified that directly or indirectly restore p53 functions and reverse progression of preneoplastic lesions and uncontrolled tumor growth [22–27]. Two widely studied p53 SMWC are CP-31398 and PRIMA-1. In particular, CP-31398 has been shown to effectively reduce tumor initiation and progression using cultured human cells *in vitro,* including germ line p53 deficient Li – Fraumeni syndrome (LFS) cells and primary mouse tumor model systems *in vivo* [28–31]. Most importantly, these agents were efficacious against a wide range of various types of tumor cells expressing mutant or wild type (WT) p53, as well as p53 null tumor cells, suggesting that direct as well as indirect mechanism(s) might account for their effect on p53. The mechanism(s) of action of these p53 modulators is being extensively investigated [32–35].

Treatment of tumors with multiple independent modalities appears to yield beneficial anti-tumor responses. Therefore, a preventative/therapeutic approach to target p53 by combining p53 SMWC together with a p53 peptide-pulsed, dendritic cell (DC)-based vaccine in a methylcholanthrene (MCA) - induced primary murine tumor model was investigated. In a previous study involving the MCA tumor model, immunotherapy with the single epitope p53_158-166_ peptide-based vaccine, p53 V1, was found to have limited efficacy due to vaccine-induced immunoselection of epitope loss variants and tumor escape [36]. Here, we posit that a p53 peptide-based vaccine combined with p53 SMWC would prove to be more effective than either modality alone for the prevention/therapy of tumors in MCA mice. The main objectives of this study were to evaluate the effect of p53 SMWC on CSC *in vitro* and the application of a combinatorial approach using p53 SMWC and p53-based vaccines to control CSC in MCA mice.

## RESULTS

### Human carcinoma cell lines sensitivity to p53 SMWC

**Figure 1:**
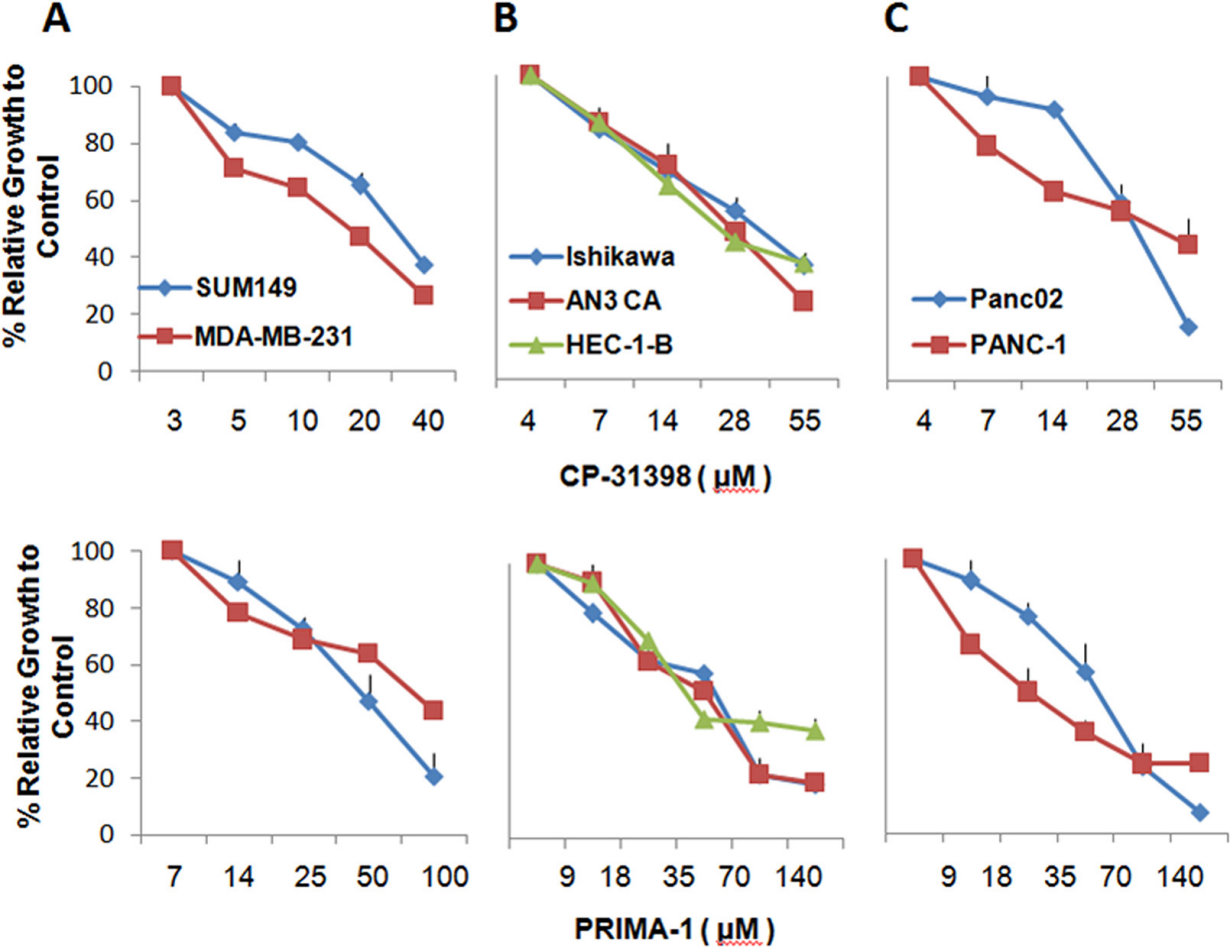
Growth of human carcinoma cell lines inhibited by p53 SMWC The effects of CP31398 and PRIMA1 on growth of a panel of human carcinoma cell lines were examined after culture for 24 h by MTT assays. Results were used to determine IC40 of each agent on the cell line. **Panel A.** SUM149 and MDA-MB-231 breast carcinoma cell lines; **Panel B.** AN3 CA, Ishikawa and HEC-1-B endometrial carcinoma cell lines; **Panel C.** PANC-1 and Panc02 cell lines.

First, the sensitivity of a panel of human tumor cell lines comprised of two breast, three endometrial, and two pancreas carcinoma cell lines to CP-31398 and PRIMA-1 was investigated. All six cell lines tested express mutant p53, with the exception of Pan02, which expresses WT p53. The cell lines were cultured in the presence of CP-31398 at 0-55 μM or PRIMA-1 at 0-140 μM (Figure 1 panels A-C). The concentrations for IC50 and maximum cytotoxicity (IC70-90) of the two p53 SMWC for all six cell lines are listed in Table 1. The CP-31398 toxicity levels for breast and endometrial carcinoma cell lines was in the range of 20-30 μM, whereas the toxicity levels for the pancreatic carcinoma cell lines was noticeably higher; range of 40-55 μM. As for PRIMA-1, the IC50 dose for all six cell lines was in the range of 35-75 μM which is higher than that for CP-31398, and where the endometrial carcinoma HEC-1-B and pancreatic carcinoma PANC-1 cell lines were the most sensitive to PRIMA-1. Further, the concentrations required for maximum cytotoxicity of all six cell lines were lower for CP-31398 (55-110 μM) than for PRIMA-1 (100-200 μM).

**Table 1:** Sensitivity of human carcinoma cell lines to p53 SMWC

Cell lines	CP-31398		PRIMA-1	
	IC50 (μM)	The concentration of maximum toxicity (μM)	IC50 (μM)	The concentration of maximum toxicity (μM)
SUM149	30	80 - IC85*	50	100-200 - IC80**
MDA-MB-231	20	80 - IC85*	75	200 - IC80*
Ishikawa	30	110 - IC80*	60	100-200 - IC80**
AN 3CA	28	55 - IC80*	50	100-200 - IC80**
HEC-1-B	28	55-110 - IC80**	40	100-200 - IC70**
Panc02	40	55 - IC85*	70	200 - IC90*
PANC-1	55	110 - IC85*	35	100-200 - IC80**

The sensitivity of a panel comprised of two human breast, three endometrial, and two pancreas carcinoma cell lines to CP-31398 and PRIMA-1. The IC50 and concentration of maximum toxicity for each carcinoma cell line were determined in standard MTT assays.

* indicates the concentration of maximum cytotoxicity following 24 h incubation with p53 SMWC.

** indicates the level of cytotoxicity remained the same in this concentration range.

### Identification of ALDH^bright^ cells by flow cytometry

**Figure 2:**
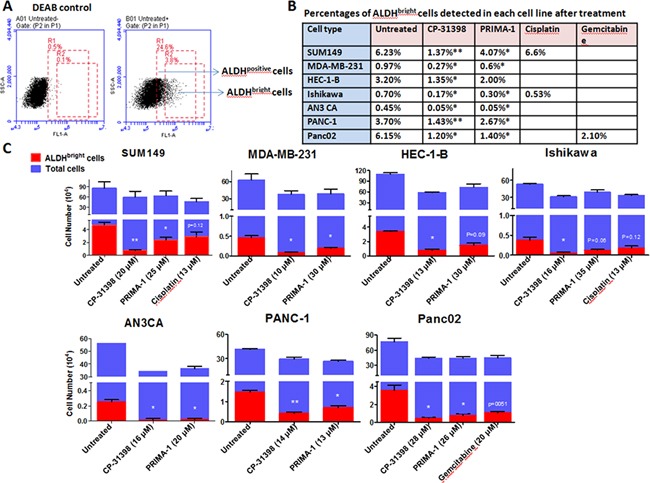
ALDH^bright^ cells in human carcinoma cell lines are sensitive to p53 SMWC Human carcinoma cells were seeded in 6-well plates and cultured overnight, followed by treatment with p53 SMWC for an additional 24 h. The selected dose of each agent was based on IC40 as determined in MTT assays. **Panel A.** example of flow cytometry analysis of a human cell line for defining the ALDH^positive^ and ALDH^bright^ cells subsets; **Panel B.** percentages of ALDH^bright^ cells detected in untreated and treated human carcinoma cell lines; **Panel C.** the ALDH^bright^ cell numbers detected in untreated and p53 SMWC-treated human carcinoma cell lines. Asterisk (*) indicates *P* < 0.05 between treated and untreated groups; asterisks (**) indicate *P* < 0.01 between treated and untreated group.

Here we focused on a subpopulation of tumor cells identified by flow cytometry that expresses 2X the mean fluorescence intensity (MFI) of bulk ALDH^positive^ cell population within a given tumor cell line, referred to as ALDH^bright^ cells. This approach is based on our previous demonstration that flow cytometry sorted ALDH^positive^ cells contain ≤ 65% ALDH^positive^ cells, with the remaining cells being ALDH^negative^ cells. In contrast, sorted ALDH^bright^ cells consist of ≥ 95% ALDH^positive^ cells (Figure 2, panel A) [19]. Most importantly, human ALDH^bright^ cells were shown to be highly tumorigenic with < 100 cells able to form human tumor xenografts in immunocompromised mice, whereas ∼ 500 to 1000 sorted ALDH^positive^ cells were required to establish tumor xenografts.

### Treatment of human carcinoma cells with p53 SMWC affects ALDH^bright^ cells content

Here we used an IC40 cutoff that was established based on the growth inhibition assays of bulk tumor cell population (Figure 1). This was done because ALDH^bright^ cells are considerably more sensitive to p53 SMWC and sufficient number of cells was required for subsequent flow-based analyses. Exposure of all six human carcinoma cell lines to CP-31398 at IC40, significantly reduced their ALDH^bright^ cell content, whether expressed as percentage or as absolute numbers (Figure 2, panels B and C). PRIMA-1 also significantly reduced the ALDH^bright^ cell content of these cell lines with the exception of HEC-1-B endometrial carcinoma cell line, which showed a noticeable, but not a significant (*P* < 0.09) reduction in the ALDH^bright^ cell content (Figure 2, panels B and C). In this regard, ALDH^bright^ cells from all cell lines showed a nearly 2X greater sensitivity to CP-31398 compared to PRIMA-1. Yet, both p53 SMWC were more effective against ALDH^bright^ cells than cisplatin when tested against the human breast carcinoma cell line SUM149 and endometrial carcinoma cell line Ishikawa, both expressing mutant p53, or gemcitabine when tested against the pancreas carcinoma cell line Pan02 expressing WT p53 (Figure 2 panel B and C).

### P53 SMWC inhibit sphere formation by human carcinoma cell lines

**Figure 3:**
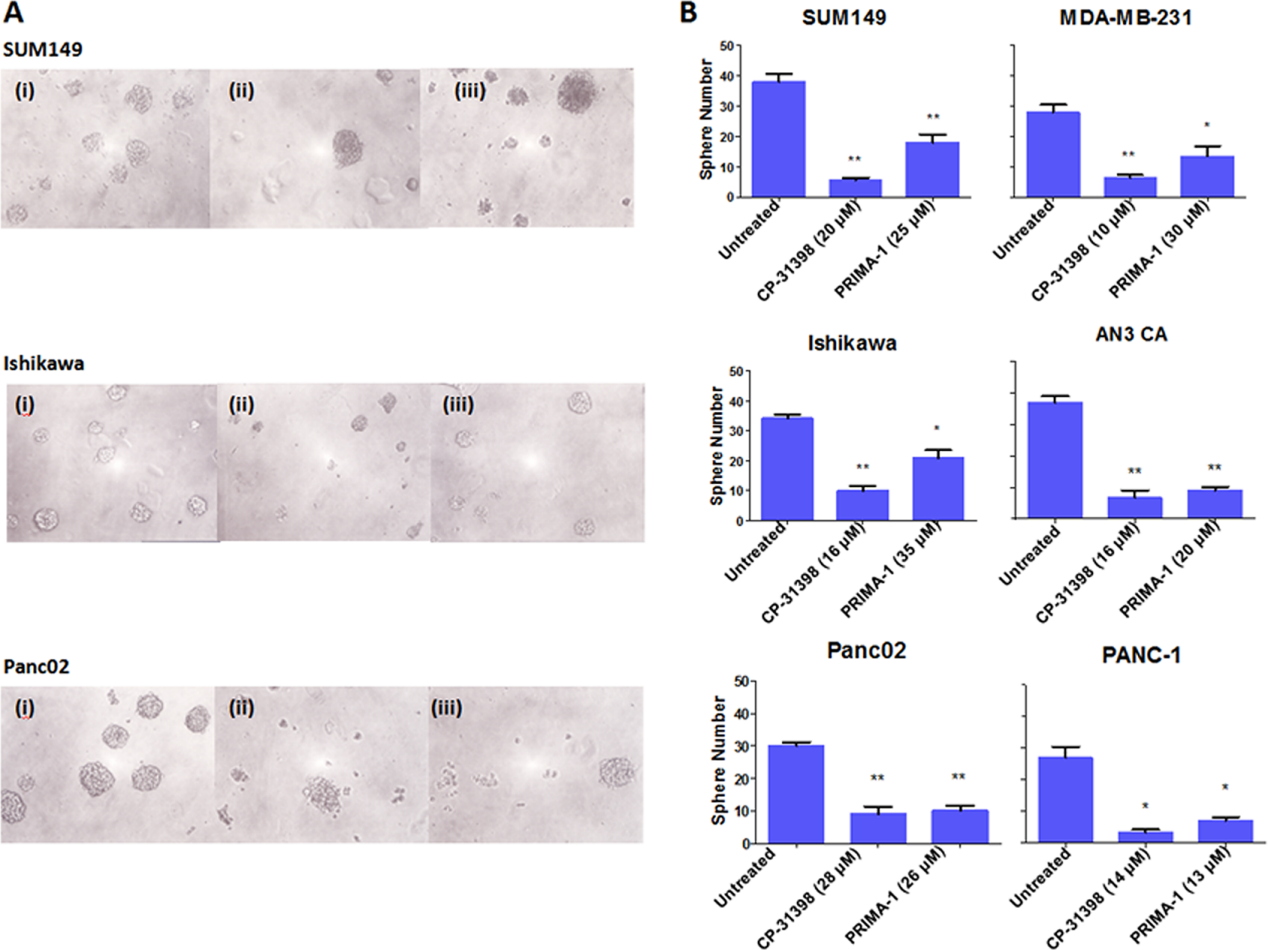
Sphere formation ability of human carcinoma cell lines inhibited by p53 SMWC The effects on the sphere formation ability of human carcinoma cell lines following treatment with CP31398 and PRIMA1, as detailed in Materials and Methods, are shown. **Panel A.** sphere formation on day 12: (i) untreated, (ii) CP31398, and (iii) PRIMA-1. **Panel B.** number of spheres in untreated and treated with either CP31398 or PRIMA-1, as indicated. Asterisks (*) indicates *P* < 0.05: treated group vs. untreated; (**) indicates *P* < 0.01: treated groups vs. untreated.

As sphere formation is a key characteristic of CSC [1], the two p53 SMWC were tested at their IC40 dose for an effect on sphere formation. Exposure to either p53 SMWC significantly reduced sphere formation by all six human carcinoma cell lines tested (Figure 3). Here as well, CP-31398 appeared to show a more pronounced inhibition of sphere formation (*P* < 0.01) compared with PRIMA-1 (*P* < 0.05) (Figure 3 panel A). However, no apparent differences in sphere sizes following exposure to CP-31398 or PRIMA-1 were visually noticeable (Figure 3 panel B).

### Combinatorial intervention of MCA mice with p53 SMWC and the p53 V1 vaccine

The efficacy of combining SMWC with a p53-based vaccine for prevention/therapy of MCA-treated mice was evaluated using a 60 d treatment protocol starting 30 d post MCA challenge (Protocol No.1). Median tumor-free survival time was used to determine the efficacy of intervention. The p53 SMWC were administered at 350 ppm in the diet continuously for 60 d, while the p53_158-166_ peptide-pulsed, DC-based vaccine, p53 V1, vaccine was administered every 10 days for a total of six immunizations over the 60 d course of treatment (schema in Supplemental Figure 1). Preliminary experiments using a lower dosage of 175 ppm of each agent alone [28–31] or in combination with the V1 vaccine failed to show efficacy.

The results in Protocol No.1 showed a modest increase in tumor-free survival times of CP-31398-treated MCA mice compared with untreated MCA mice (127 ± 15 d vs. 113 ± 3 d *P* < 0.194) (Table 2), while treatment with PRIMA-1 only marginally enhanced their tumor-free survival (120 ± 31 d, *P* < 0.736) compared with control mice. Interestingly, treatment of mice with the p53 V1 vaccine alone decreased their tumor-free survival (106 ± 10 d). When either CP-31398 or PRIMA-1 were administered together with the p53 V1 vaccine, there was a more noticeable, but not significant enhancement of survival compared to control mice (134 ± 18 d vs. 113 ± 3 d; *P* < 0.089, and 127 ± 4 d vs. 113 ± 3 d; *P* < 0.138, respectively). In this regard, tumor incidence was not impacted by any of these interventions wherein tumors were seen in 10/10 mice in each group.

**Table 2:** Survival analysis of Protocol No. 1 MCA mice treated with p53 SMWC and p53 V1 vaccine

Group	No. mice	p53 SMWC	dosage	p53 vaccine	Frequency of immunization	Median tumor-free survival ± MSE, days	Log Rank (Mantel-Cox) significance
1	10	none		none		113 ± 3	
2	10	CP-31398	350 ppm	none		127 ± 15	0.194
3	10	PRIMA-1	350 ppm	none		120 ± 31	0.736
4	10	none		V1	6X	106 ± 10	0.637
5	10	CP-31398	350 ppm	V1	6X	134 ± 18	0.089
6	10	PRIMA-1	350 ppm	V1	6X	127 ± 4	0.138

MCA micewere treated starting at 30 d post MCA challenge for 60 d continuously with p53 SMWC and p53 V1 vaccine as detailed in Materials and Methods. Median tumor-free survival and Log-Rank significance of survival times of groups of MCA mice are listed. Group 4 mice, which were treated with CP-31398 and p53 V1 vaccine, had a notable but not significant increase in tumor-free survival compared to untreated MCA mice.

Abbreviations: MCA, methylcholantrene; SMWC, small molecular weight compound; V1, the p53_158-166_ peptide-pulsed, DC-based vaccine.

Taken together, these results show that treatment with p53 SMWC alone failed to significantly enhance tumor-free survival of MCA-treated mice. As for treatment with the single epitope p53 V1 vaccine, its lack of efficacy was also noted in our previous study and was attributed to immunoselection and the outgrowth of vaccine-induced epitope loss tumors [36]. The tumors either expressed mutations within the targeted p53 peptide epitope or have undergone a loss of H-2K^b^ expression, which abrogated the ability of T-cell effectors to target these tumors [36]. It is of interest that intervention with either p53 SMWC in combination with the p53 V1 vaccine improved tumor-free survival compared to vaccine alone, suggestive of a negative interaction between these two modalities that impacted p53 V1 vaccine-induced immunoselection and enhanced survival.

### Sensitivity of tumor cells from p53 SMWC-treated MCA mice to p53 SMWC

A possible explanation for the modest effect of p53 SMWC on tumor-free survival in MCA mice is the outgrowth of p53 SMWC resistant tumor cells in these mice. Therefore, low passage cell lines derived from tumors harvested from p53 SMWC-treated MCA mice were investigated for *in vitro*-sensitivity to the p53 SMWC. The results show that the cell lines isolated from tumors of CP-31398- or PRIMA-1-treated MCA mice (MCA1.5 and MCA 5.5, respectively) were as sensitive to these agents as were cells isolated from a tumor of untreated MCA mouse (MCA 17.4 cell line) (Table 3).

**Table 3:** *In vitro* sensitivity to p53 SMWC of ALDH^bright^ cells present in tumor cell lines derived from tumors of MCA mice treated with p53 SMWC

Tumor cell lines	Untreated control		CP-31398 treated			PRIMA-1 treated		
	ALDH^+^ cells	ALDH^bright^cells	Bulk cells	ALDH^+^ cells	ALDH^bright^cells	Bulk cells	ALDH^+^ cells	ALDH^bright^ cells
MCA 17.4 (untreated)	2.3%	0.03%	−80%	−95%	−95%	−68%	−85%	−66%
MCA 1.5 (CP-31398)	3.1%	0.09%	−75%	−88%	−66%	−73%	−75%	−70%
MCA 5.5 (PRIMA-1)	3.0%	0.96%	−71%	−83%	−75%	−75%	−95%	−90%

Low passage cell lines (≤ 3) derived from tumors harvested from p53 SMWC-treated MCA mice were analyzed for sensitivity to treatment with CP-31398 (20 μM) and PRIMA-1 (32 μM). In addition to determining cytotoxicity to the bulk population of tumor cells, the treated cells were analyzed for ALDH^positive^ and ALDH^bright^ cell content.

Abbreviations: MCA, methylcholantrene; SMWC, small molecular weight compound.

### Frequency of p53_158-166_ peptide-specific CD8^+^ T cells in MCA mice treated with p53 SMWC and p53 V1 vaccine

**Figure 4:**
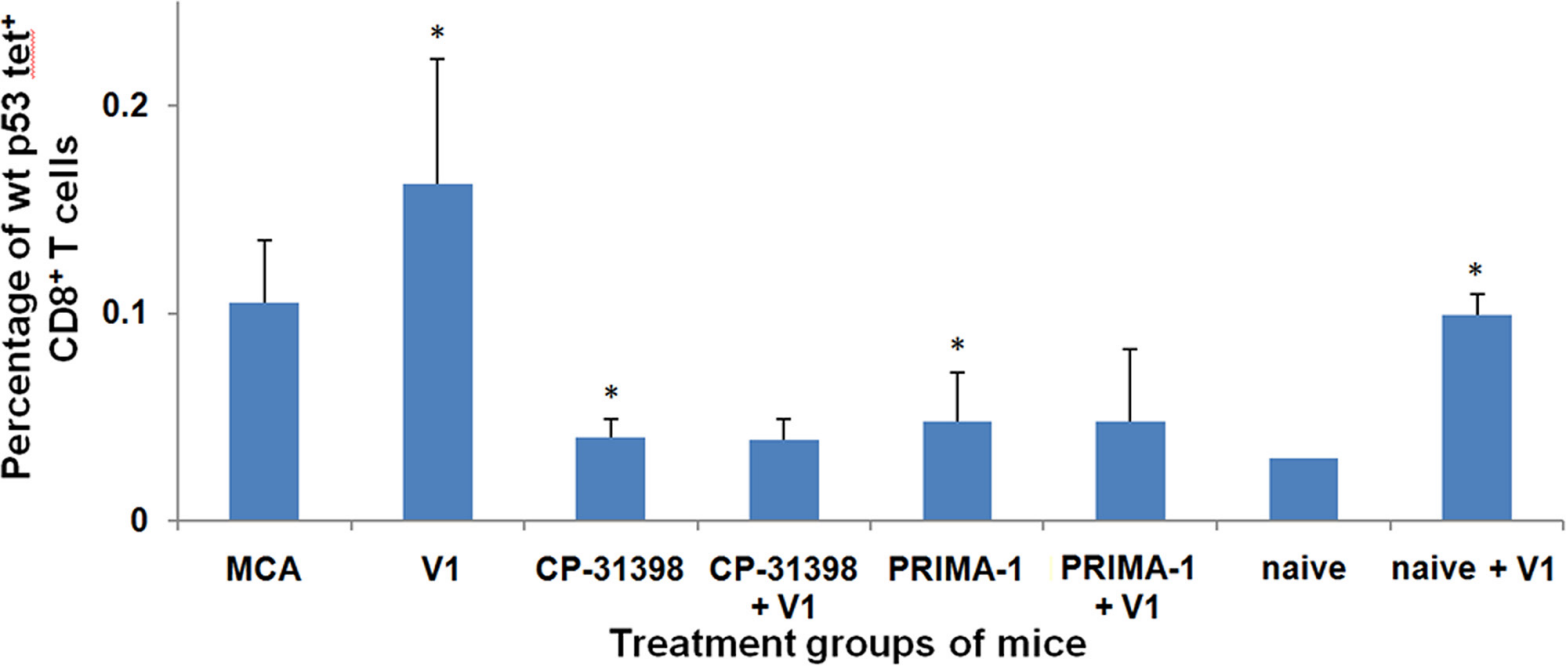
p53 peptide-specific CD8+ T-cell responses of Protocol No.1 MCA mice Splenocytes of treated and untreated MCA mice were analyzed by flow cytometry using H2-K^b^/p53_158-166_ peptide tetramer complexes to determine the percentages of CD8^+^ T cells that were H2-K^b^-restricted, p53_158-166_ peptide-specific CD8^+^ T cells. Asterisk (*) indicates significant (*P* < 0.05) downregulation in treated groups of MCA mice compared to untreated MCA mice or enhancement in naive + p53 V1 mice compared to naive control mice.

Previously, we reported that during the initial 30 d-60 d post MCA challenge period, the level of p53_158-166_ peptide-specific CD8^+^ T cells was elevated compared to that detected in naive mice [36]. In contrast, the results in Protocol No. 1 showed that the frequency of p53_158-166_ peptide-specific CD8^+^ T cells in MCA mice administered either CP-31398 or PRIMA-1 with the p53 V1 vaccine was significantly down-regulated compared with control MCA mice (Figure 4). Thus, while the p53 V1 vaccine alone increased the frequency of p53_158-166_ peptide-specific CD8^+^ T cells, this was not seen when the vaccine was combined with either p53 SMWC. These findings clearly point to a potential incompatibility between the p53 SWMC and vaccine modalities of the combined intervention.

### Sensitivity of activated mouse and human immune cells to p53 SWMC

**Figure 5:**
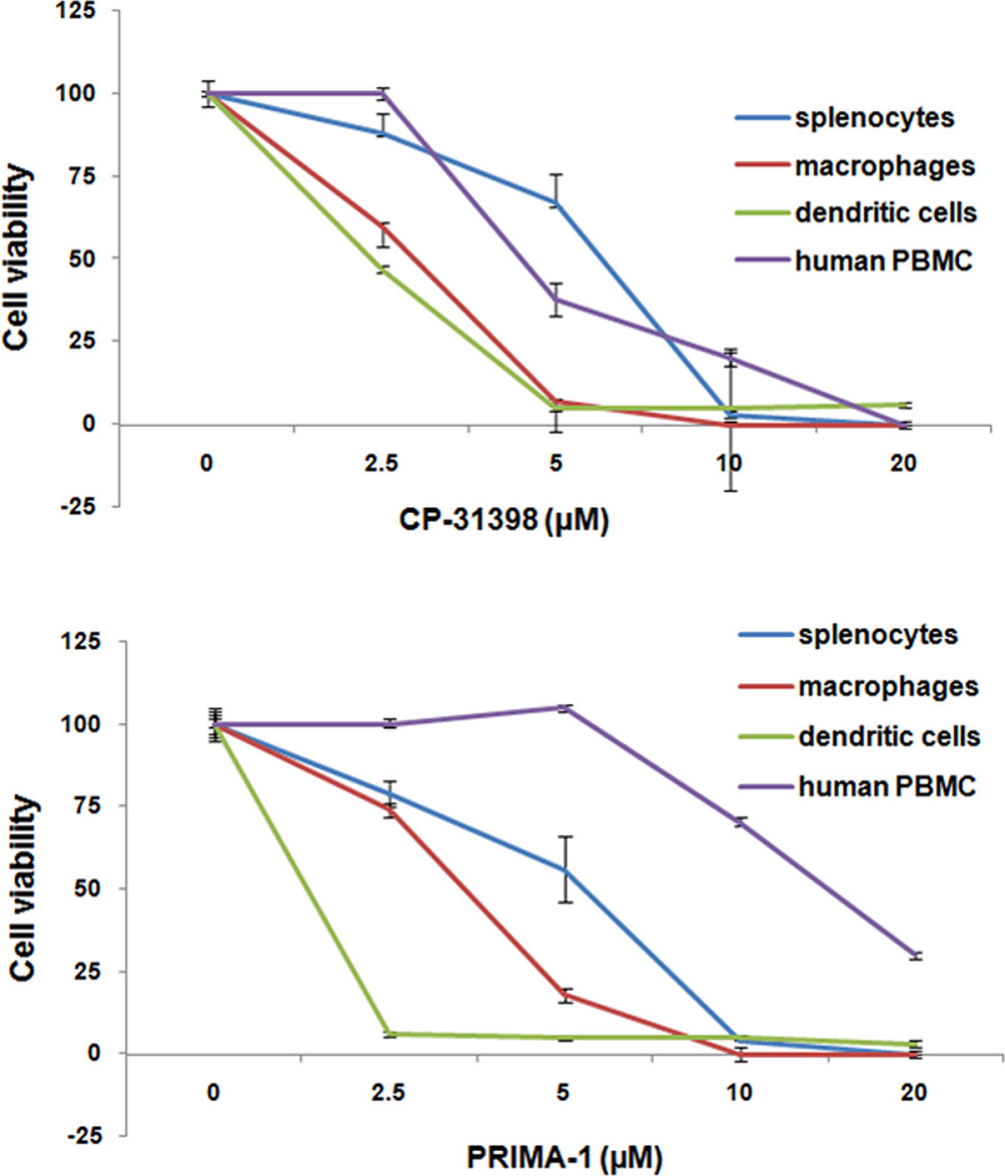
*In vitro*-sensitivity of activated mouse and human lymphocytes to p53 SMWC Viability of Con A-activated mouse splenocytes, rmIFNγ activated mouse macrophages, bone marrow-derived mouse dendritic cells, and PHA-activated PBMC following activation in the presence of p53 SWMC. See Materials and Methods for additional details.

In one of our initial descriptions of p53 we reported its enhanced expression in normal cells with high proliferative activities, including mitogen-activated T cells [37]. Therefore, the possibility that CP-31398 or PRIMA-1 were able to target activated immune cells was investigated by analyzing the *in vitro*-sensitivity of distinct populations of activated mouse and human immune cells to CP-31398 or PRIMA-1. Both p53 SMWC were found to be similarly toxic to conconavalin A (ConA)-activated mouse splenocytes, recombinant mouse interferon gamma (rmIFNγ) activated-mouse macrophages and mouse bone marrow-derived DC, as well as phytohemagglutinin (PHA)-activated human PBMC (Figure 5). The IC50 dose of CP-31398 for human and mouse mitogen-activated immune cells was in the range of 5-10 μM (Figure 5). In contrast, the IC50 of PRIMA-1 for these immune cell populations showed greater variation with ∼ 10 μM for DC, ∼ 20 μM for macrophages, ∼ 25 μM for splenocytes and ∼ 75 μM for human PBMC. Nonetheless, the overall sensitivity of activated immune cells to the p53 SMWC was not substantially different from IC50 seen with the human carcinoma and mouse tumor cell lines tested (Figure 1 and Table 3). These results suggest that p53 SMWC-induced activation of endogenous p53 in cells already enriched for p53 expression is shared by both primed immune cells and tumor cells, presumably leading to their toxic response to p53 SMWC, which is likely manifested as induced senescence. The sensitivity of DC to the p53 SMWC further indicates a marked incompatibility of the SMWC and the vaccine vehicle used in the combinatorial treatment of MCA mice.

### Combinatorial intervention of MCA mice with p53 SMWC and p53 V2 vaccine

In order to enhance the anti-tumor effect of p53 SMWC in the MCA mouse model, a second protocol was initiated which differed from Protocol No.1 in several key respects. In Protocol No.2, the intervention period started at d30 post challenge but was extended from 60 d to 90 d, and included only CP-31398. In order to minimize the apparent incompatibility seen in Protocol 1 regarding the application of p53 SMWC with the p53 V1 vaccine, these two modalities were administered separately in two sequential 45 d periods. Lastly, here we used the multiepitope p53 peptide-pulsed, DC-based vaccine, p53 V2, that potentially was expected to yield a more robust anti-p53 immune response than the single epitope p53 V1 vaccine (schema in Supplementary Figure S2).

Analysis of tumor-free survival showed that treatment with CP-31398 for the entire 90d period, noticeably but not significantly enhanced survival of MCA mice compared to control (120 ± 15.5 d vs. 100 ± 7.7 d; *P* < 0.131), a finding comparable to that obtained in Protocol No 1 (Table 4). However, in contrast to results obtained with the p53 V1 vaccine, administration of the p53 V2 vaccine to MCA mice significantly prolonged their tumor-free survival compared to control MCA mice (150 ± 10.5 d vs. 100 ± 7.7 d; *P* = 0.001).

**Table 4:** Survival analysis of Protocol No. 2 MCA mice treated with p53 SMWC and p53 V2 vaccine

Group	No. mice / group	Therapy d45-d90	Dosage SMWC and / or frequency of immunization	Therapy d90-d135	Dosage SMWC and / or frequency of immunization	Median tumor-free survival ± MSE days	Log Rank (Mantel-Cox) significance
1	10	none		none		100 ± 7.7	
2	10	CP-31398	350 ppm	CP-31398	350 ppm	120 ± 15.5	0.131
3	10	V2	3X	V2	3X	150 ± 10.5	0.001
4	10	CP-31398 + V2	350 ppm + 3X V2	CP-31398 + V2	350 ppm + 3X V2	130 ± 10.5	0.049
5	10	CP-31398	350 ppm	V2	3X	120 ± 9.3	0.075
6	10	V2	3X	CP-31398	350 ppm	120 ± 7.8	0.048

MCA micewere treated starting at 30 d post MCA challenge for 90 d continuously with p53 SMWC and p53 V2 vaccine or sequentially for 45 d periods as indicated. See Materials and Methods for further details. Median tumor-free survival and Log-Rank significance of survival time

Abbreviations: MCA, methylcholantrene; SMWC, small molecular weight compound; V2, the multiepitope p53 peptide-pulsed, DC-based vaccine.

Administration of CP-31398 with p53 V2 vaccine throughout the 90d treatment period was significantly effective (130 ± 10.5 d vs. 100 ± 7.7 d; *P* = 0.049), but not as effective as treatment with p53 V2 vaccine as monotherapy. However, sequential 45 d administrations of CP-31398 with the p53 V2 vaccine, regardless of which modality was administered first, showed no improvement in the median tumor–free survival of 120 d achieved by administering CP-31398 alone. Nonetheless, the results did indicate that when a sequence of p53 V2 vaccine followed by CP-31398 is used, but not the reverse, there was a positive and significant effect on tumor-free survival (*P* < 0.048 vs. *P* < 0.075).

Relative to tumor incidence, p53 V2 vaccine was the most effective treatment. Tumors appeared in only 4/10 mice administered the p53 V2 vaccine compared with a tumor incidence of 8 or 9/10 mice in all other groups of MCA treated mice in Protocol No.2.

### Frequency of p53_158-166_ peptide-specific CD8^+^ T cells in MCA mice treated with p53 SMWC and p53 V2 vaccine

**Figure 6:**
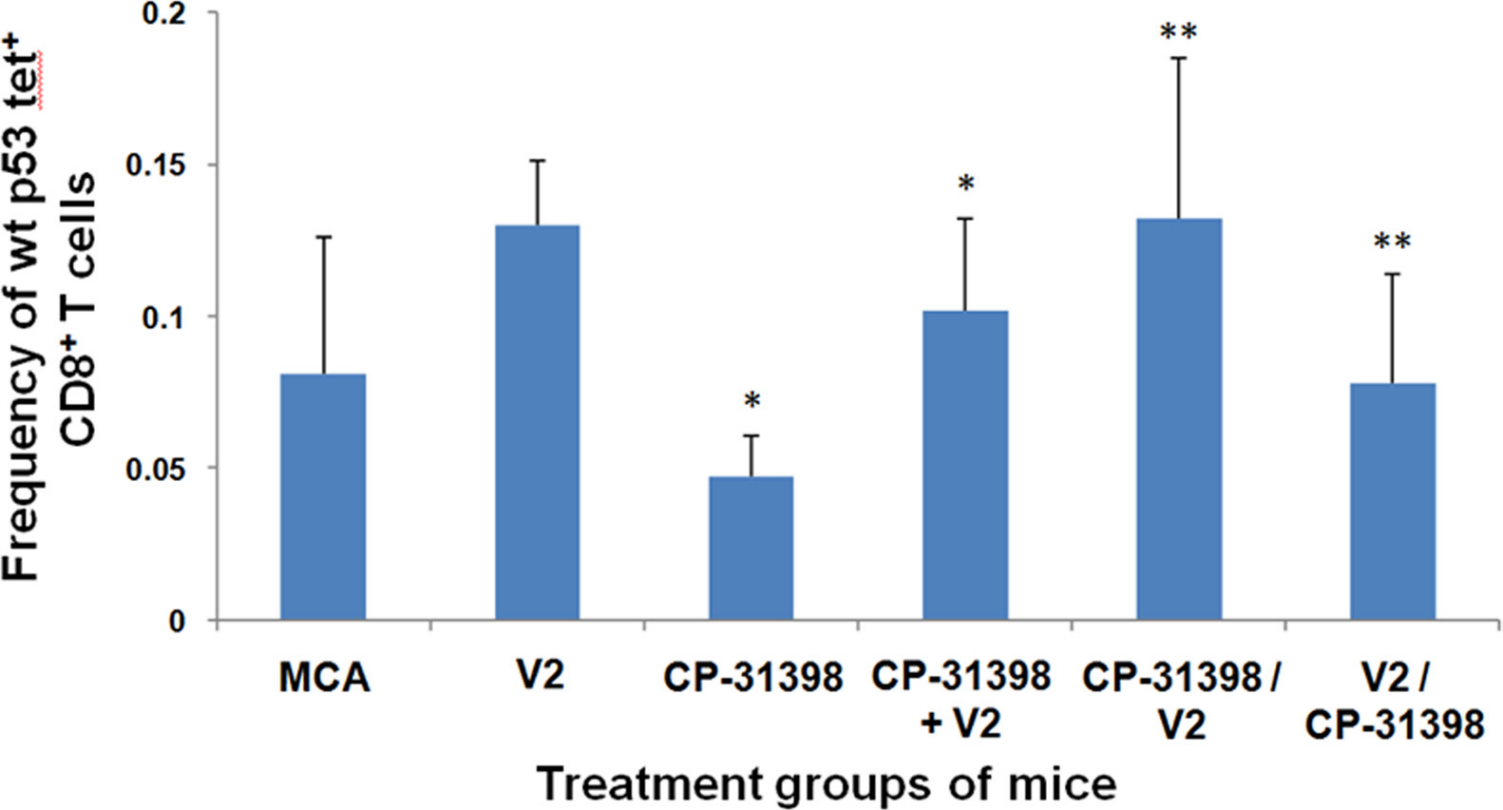
p53 peptide-specific CD8^+^ T-cell responses of Protocol No 2 MCA mice. Splenocytes of treated and untreated MCA mice were analyzed by flow cytometry using APC-conjugated-H2-K^b^/p53_158-166_ peptide tetramer complexes to determine the percentages of CD8^+^ T cells that were H2-K^b^-restricted, p53_158-166_ peptide-specific CD8^+^ T cells. Asterisks (*) indicates significance (*P* < 0.05) compared to untreated MCA control mice, (**) indicate significance (*P* < 0.05) relative to CP-31398 treated mice.

Again, using p53_158-166_ peptide-specific T-cell effectors as a surrogate marker, our results using Protocol No. 2 showed that CP-31398 significantly decreased the percentage of p53_158-166_ peptide-specific CD8^+^ T cells in MCA mice compared to control mice (Figure 6). In contrast, immunization of MCA mice with p53 V2 vaccine alone increased the frequency of anti-p53 effectors. Furthermore, the combinatorial intervention of CP-31398 and p53 V2 vaccine, whether continuous or sequential, significantly increased the frequency of anti-p53 T- cell effectors compared with control or CP-31398-treated mice, but not compared with mice receiving only the p53 vaccine V2. Thus, use of the more robust p53 V2 vaccine attenuated the negative impact that CP-31398 had on p53-specific T-cell effectors in MCA mice.

## DISCUSSION

The rationale for targeting p53 is its near universal loss of function during cancer development. As such, p53 SMWC join a selected group of SMWC capable of effectively targeting CSC [5]. Yet the fact that p53 plays a pivotal role in multiple pathways during normal and cancer cell development likely contributes to complex interactions through which CP-31398 or PRIMA-1 function. For example, as shown here, p53 SMWC were efficacious against all tumor cells *in vitro* regardless of p53 status, whether WT or mutant, indicating that a direct interaction with an altered p53 molecule does not necessarily represent their exclusive mode of action. Nonetheless, recent experiments involving mutant p53-driven Li-Fraumeni cells, clearly demonstrate that the effect of these p53 SMWC is largely due to their direct impact on p53 (32–35, 38). For this reason, a combinatorial approach comprised of a p53 SWMC and a p53-specific immune-intervention may provide increased efficacy, particularly against ALDH^bright^ cells.

Our results clearly demonstrate the ability of CP-31398 and PRIMA-1 to inhibit ALDH^bright^ CSC cells *in vitro*, including the inhibition of sphere formation by these cells. However, *in vivo*-based experiments with p53 SMWC agents showed that they had only a noticeable but not significant impact on tumor-free survival in the MCA mouse tumor model. This lack of efficacy in the MCA model is in marked contrast to the well-documented efficacy of these p53 SMWC in several other primary murine tumor model systems [28–31]. Importantly, their modest effect *in vivo* on MCA mice was not due to the outgrowth of p53 SMWC resistant tumor cells, since our *in vitro*-based analysis indicated that early passage cell lines derived from tumors induced in these mice were highly sensitive to p53 SMWC.

Two different mechanisms that are not unrelated may account for this discrepancy; one is specifically based on the MCA mouse model, the other carries wider implications related to the potential effects of p53 SMWC on host immune response. The MCA mouse model is characterized by tumors uniformly expressing mutant p53 in response to MCA, a polycyclic aromatic hydrocarbon (PAH). Recently, mutant p53 has been shown to up regulate aryl hydrocarbon receptor (AHR) signaling via its effect on the Aha-1/HSP90/ATPase axis, leading to enhanced expression of xenobiotic response elements, including CYP1A1/CYP1B1 and a significant increase of PAH conversion to the active carcinogen, concomitant with a sustained activation of the Wnt pathway [34]. Therefore, the potential increase of an active proximal carcinogen, including global activation of tumor associated pathways, might have affected at many levels the ability of p53 SMWC to significantly inhibit tumor induction and growth in MCA mice. Further studies are warranted to investigate possible direct and/or indirect molecular interactions between p53 SMWC agents and MCA and MCA-induced alterations that might have influenced the reduced efficacy of the agents.

Along these lines, administration of p53 SMWC together with a p53 peptide-based vaccine did not show a synergistic effect on survival of the MCA mice. In addition, while the more robust p53 V2 vaccine was effective in enhancing survival when administered as a monotherapy, its combination with CP-31398 was weak in these mice. In retrospect, these results provide insights into potential pharmacodynamic effects of p53 SMWC that were not evident in previously based *in vivo*-based studies involving these agents. Thus, the initial identification of p53 as a transformation-related antigen described its detection at elevated levels in chemical, radiation and viral-induced mouse tumors, as well as in normal mouse tissues and cells having high proliferation rates, in particular, mitogen-activated lymphocytes [37, 39, 40]. Our analysis of splenic p53_158-166_ peptide-specific T cells from MCA-treated mice showed that p53 SMWC, particularly when administered together with p53 peptide-based vaccines, decreased the frequencies of these T-cell effectors in treated mice and that this effect was quite pronounced when the single epitope p53 V1 vaccine was employed. Notably, our *in vitro* analysis further demonstrated that these p53 SMWC agents were capable of blocking the activation of human and mouse immune cell populations, including activated lymphocytes as well DC, the vaccine vehicle. One can presume, therefore, that as part of their mode of action, p53 SMWC further activate “normalized” and constitutively present WT p53 at a sustained level, leading to senescence of effector cells that are essential for a robust immune response. These results suggest that dose recalibration of p53 SMWC, whether administered alone or in the presence of vaccine, may be necessary.

In conclusion, this study points to the following important findings. First, CP-31398 and PRIMA-1 are able to eliminate CSC in human-derived tumor cell lines *in vitro*. Second, cells expressing WT and mutant p53 were similarly affected by these p53 SMWC *in vitro*. Third, p53 SMWC are considerably more effective against CSC *in vitro* compared with two often-used chemotherapeutic agents, namely cisplatin and gemcitabine. A fourth important observation is that the effect of p53 SMWC on activated immune cell populations was associated with diminished anti-p53 immune response in MCA-treated mice. Presumably this might be due to activation and sustained expression by p53 SMWC of p53 in activated immune cells, leading to their reduced survival and a negative impact on immune responses. Nonetheless, the effect of p53 SMWC on CSC is of great interest [41]. However, development of p53 SWMC that exclusively target mutated p53 molecules, including dose calibration should minimize their impact on the host immune system and enhance their clinical efficacy in cancer prevention/therapy.

## MATERIALS AND METHODS

### P53 modulatory SMWC and other agents

CP-31398 (Cat. No. PZ0115) and PRIMA-1 (Cat.No. P0069) were purchased from Sigma Chemical Co., St, Louis, MO. Gemcitabine was obtained from Zydus Hospira Oncology Private Ltd., Ahmedabad, Gujarat, India, and cisplatin from Jiangsu Hansen Pharmaceutical Co., Ltd., Jiangsu, China. RmIFNγ was purchased from R&D Systems, Minneapolis, MN, and ConA, PHA and MCA from Sigma.

### Human carcinoma and mouse tumor cell lines

The human breast carcinoma cell lines MDA-MB-231 and SUM149 were acquired from Duke Comprehensive Cancer Center Cell Culture Facility (Durham, NC 27710). The human and mouse pancreatic ductal adenocarcinoma cell lines, respectively, PANC-1 and Panc02, as well as the endometrial carcinoma HEC-1-B (ATCC® HTB-113) and AN3CA (ATCC® HTB-111D) cell lines were obtained from the American Type Culture Collection (ATCC), Rockville, MD. The endometrial carcinoma cell line, Ishikawa, was obtained from Sigma. All the cell lines used in this study with the exception of PANC-1 were cultured using a complete media (CM) consisting of RPMI-1640 supplemented with 10% FBS and L-glutamine (Mediatech, Inc Westwood, MA). The PANC-1 cell line was maintained in DMEM medium supplemented with 10% FBS, 4.5 g/L glucose and L- glutamine (Mediatech, Inc). The MDA-MB-231 and SUM149 breast carcinoma cell lines express the p53 R280K and p53 M237I mutations, respectively. The pancreas carcinoma PANC-1 and Panc02 cell lines express the R273H mutation and wtp53, respectively, while the endometrial carcinoma cell lines, HEC-1-B, Ishikawa and AN3 CA express the p53 R248Q, M246V and R213Q mutations, respectively.

The mouse derived MCA 17.4, 1.5 and 5.5 tumor cell lines were established from primary tumors harvested from untreated and p53 SMWC-treated MCA mice. MCA 17.4 was obtained from an untreated MCA control mouse, MCA 1.5 from a CP-31398-treated MCA mouse and MCA 5.5 from a PRIMA-1-treated MCA mouse. Tumor specimens were minced and pieces placed in T25 flasks containing 5 ml CM until a confluent monolayer of tumor cells was established. In general, tumor cells harvested from passages < 3 were used in this study.

### Human PBMC

Blood specimens were obtained by a written consent from normal donors at the University of Pittsburgh Tissue Bank with IRB approval #980633. Peripheral blood mononuclear cells (PBMC) were isolated and stimulated with PHA using standard procedures [42].

### Mice and activated mouse immune cells

C57BL/6 J (B6/J) female mice (6 to 8 weeks of age) were obtained from The Jackson Laboratories (Bar Harbor, ME) and used under protocols approved by the Institutional Animal Care and Use Committee (IACUC) of the University of Pittsburgh. The isolated splenocytes and macrophages were activated using Con A and rmIFNγ, respectively, using standard procedures [42].

### Cell viability assay [43]

A cell viability assay using yellow tetrazolium salt3-(4,5-Dimethylthiazol-2-yl)-2,5-diphenyl-tetrazolium bromide or MTT (Sigma) wasutilized to assess the effects of the p53 SMWC on growth of human carcinoma cell lines [41]. Cells were plated in triplicate in 96-well plates at a density of 2.5 × 10^3^ cells / well in 100 μL of complete medium. After 24hr incubation in a humidified 5% atmosphere at 37°C, the cells were treated with increasing concentrations of SMWC for an additional 24 hr period and analyzed for cell growth using the MTT assay. Stock solutions (10 mM) of CP-31398 and PRIMA-1 in PBS were diluted in PBS immediately prior to use. The assay was performed as follows: a 12 mM MTT stock solution was prepared by adding 1 mL of sterile PBS to 5 mg MTT and mixing by vortex or sonication until dissolved. Once prepared, the MTT solution was stored for four weeks at 4°C protected from light. A 500 mL SDS-HCl solution consisting of 0.01 M HCl, 10% propanol (Sigma) and 5 gm SDS (Sigma) was prepared by mixing the solution gently by inversion until the SDS dissolved.100 μL of cell culture medium was removed from each well and 10 μL of the 12 mM MTT stock solution added. A negative control consisting of 10 μL of the MTT stock solution added to 100 μL of medium was prepared. The plates were incubated at 37°C for 4hr followed by the addition of 100 μL of the SDS-HCl solution to each well and mixing thoroughly using a pipette. The absorbance of each sample was read at 570 nm in an ELISA plate reader (Epoch Bio Tek, Winooski, VT). The inhibitory concentration (IC40) doses were determined using standard procedure.

### *In vitro*-treatment of human carcinoma and mouse tumor cell lines with p53 SMWC

Human tumor cell lines were plated at a cell density of 2 × 10^5^ cell/per well in 6-well plates in 2 ml of CM and incubated overnight at 37°C in a 5% CO_2_ humidified atmosphere. The cells were then cultured in the presence of CP-31398 at 0-20 μM and PRIMA-1 at 0-32 μM. After 24 hr incubation, the cells were harvested. The effects of the p53 SMWC on cell growth were calculated relative to the untreated control cell populations.

### Flow cytometry analysis of ALDH^positive^/ALDH^bright^ cells in human and mouse tumor cell lines

The p53 SMWC-treated and untreated human and mouse tumor cell lines were analyzed for ALDH^positive^/ALDH^bright^cellsby flow cytometry using ALDEFLUOR (StemCell Technologies Vancouver, BC, V5Z 1B3, Canada), as previously described [18, 19, 44]. In general, duplicate aliquots of 2 × 10^5^ tumor cell samples were incubated with ALDEFLUOR, with or without the ALDH inhibitor, diethylaminobenzaldehyde (DEAB) (control), according to the manufacturer's instructions. The control aliquot was analyzed by flow cytometry and set for detection of ≤0.5% ALDH^positive^ cells. Using this cutoff, the test aliquot was analyzed to identify its ALDH^positive^/ALDH^bright^ cell content with ALDH^bright^ cells defined as the ALDH^positive^ cells with double the mean fluorescence intensity (MFI) of the bulk population of ALDH^positive^ cells in a sample [19]. The flow cytometry analyses were performed using a C6-Sample cytometer (BD); all samples were run using identical settings to collect a minimum of 8,000-gated events. Analyses were done using BD CSAMPLER™ ANALYSIS software (BD). The effects of the p53 SMWC on cell growth and percentage content of ALDH^positive^/ALDH^bright^ cells were calculated relative to the untreated control cell populations.

### Sphere formation assay [44]

Untreated and p53 SMWC-treated human carcinoma cells were planted in triplicate wells (1 × 10^3^ cells/well) in 24-well Ultra-Low attachment plates (Corning Incorporated) with sphere formation medium (500 μL of mixed medium containing 32% MethoCult medium / 20% MammoCult basal human medium (final concentration of 2% MammoCult proliferation supplements (Stem Cell Technologies), including 48% DMEM supplemented with final concentrations of 100 pg/mL EGF, 50 ng/mL bFGF, 5 ng/mL stem cell factor, 1 μM hydrocortisone, and 5 μg/mL insulin at 37°C in a 1% O_2_ and 5% CO_2_ humidified atmosphere for 12 d. The total sphere number was the sum of the number of the spheres counted in 6 random fields of each well using a Zeiss Inverted Fluorescence Microscope at 100X magnification.

### p53 peptide-based vaccines

DC were generated from B6/J mouse bone marrow cells obtained from flushed marrow cavities of femurs and tibias as described previously [36]. Two different p53 peptide-based vaccines were used in this study, the single epitope p53 V1 and the multiepitope p53 V2 vaccines. The peptides used in this study were prepared by Drs. Ryo Hayashi and Ettore Appella. The p53 V1 vaccine employed the H2-K^b^-restricted, CD8^+^ T cell-defined p53_158–166_ (AIYKKSQHM) peptide. The p53 V2 vaccine consisted of a mixture of primarily 30 residue 15-mer overlapping p53 peptides corresponding to p53 residues 70-248. Peptide sequences are listed below. The peptides were pulsed onto B6 bone marrow derived-DC. Stock solutions consisting either of the single or the ten overlapping peptides, each at a concentration of 10 mg/ml (total peptide concentration of latter being 100 mg/ml) were prepared in DMSO and aliquots stored at −20°C. The DC were pulsed with the peptides at a concentration of 10μg/ml each peptide/PBS (1:1000 dilution of stock solution). Freshly prepared or previously stored (at −80°C) DC were pulsed for 60 min at RT at a density of 5 × 10^6^ cells/ml PBS with the peptide(s), washed and suspended at a concentration of 2 × 10^6^ cells/ml PBS before injection.

Overlapping p53 peptides:

**Table:** 

p53_70-99:_	QDPVTETPGPVAPAPATPWPLSSFVPSQKT
p53_86-115:_	TPWPLSSFVPSQKTYQGNYGFHLGFLQSGT
p53_102-131:_	GNYGFHLGFLQSGTAKSVMCTYSPPLNKLF
p53_126-155:_	PLNKLFCQLAKTCPVQLWVSATPPAGSRVR
p53_142-172:_	LWVSATPPAGSRVRAMAIYKKSQHMTEVVRR
p53_157-186:_	MAIYKKSQHMTEVVRRCPHHERCSDGDGLA
p53_174-203:_	PHHERCSDGDGLAPPQHLIRVEGNLYPEYL
p53_190-219:_	HLIRVEGNLYPEYLEDRQTFRHSVVVPYEP
p53_206-235:_	RQTFRHSVVVPYEPPEAGSEYTTIHYKYMC
p53_221-248:_	EAGSEYTTIHYKYMCNSSCMGGMNRRPI

### Immunization of mice

Groups of B6/J mice were immunized with 2 × 10^5^ p53 peptide-pulsed DC injected subcutaneously (s.c) at 7 d, 10 d or 14 d intervals, depending on the protocol. Each immunization consisted of s.c. injection of 50μl of a suspension of 2 × 10^6^ p53 peptide-pulsed DC/ml PBS in both inguinal node regions. Splenocytes are harvested from mice and analyzed by flow cytometry for their frequency of H2-K^b^-restricted, p53_158–166_ peptide-specific CTL using H2-K^b^/wt p53_158-166_ peptide tetramers.

### H2-K^b^-restricted, p53_158-166_ peptide-specific tetramer^+^ CD8^+^ T cells [36]

Soluble allophycocyanin conjugated-H2-K^b^/p53_158-166_ peptide tetramer complexes prepared by the NIH Tetramer Facility (Atlanta, GA) and FITC-anti-mouse CD8 clone CT-168F (Accurate Chemical, Westbury, NY) monoclonal antibody were used to detect splenic H2-K^b^ restricted, p53_158-166_ peptide-specific CD8^+^ T cells, as previously described. All the flow cytometry analyses were performed using an FC500 cytometer (Beckman Coulter); all samples were run using identical settings to collect a minimum of 10,000 gated events, when possible. Analyses were performed using EXPO32 ADC software (Beckman Coulter) or Summit V4.3 (Dako).

### MCA-induced mouse tumor model

The animal protocols used in this study were reviewed and approved by The Institutional Animal Care and Use Committee of the University of Pittsburgh as meeting the standards for animal care and use set by the Animal Welfare Act and the NIH Guide for the Care and Use of Laboratory Animals. B6/J female mice were injected s.c. with a bolus of MCA [1 mg/ml sesame oil (Sigma)], which was prepared immediately prior to use. Within 30d of challenge, nodules at sites of injection are palpable and progressively grow into tumors (≥ 5 mm diameter) within 50-90 d post challenge. Studies involving MCA - challenged mice terminated on or about 150 d post challenge as all the mice have tumors exceeding allowed tumor size limits, necrotic tumors and/or show decreased health status mandating their removal from a study at this time point.

Two distinct experimental protocols were used to evaluate the efficacy of the combinatorial intervention using p53 SMWC continuously administered at 350 ppm of diet alone with either the p53 V1 or p53 V2 vaccine (total of 6 immunizations). Both started at d30 post challenge. Protocol No.1 consisted of administration for a 60 d period of either CP-31398, PRIMA-1 or p53 vaccine V1 alone or in combination with each other (Supplementary Figure S1). Protocol No.2 consisted of administration of CP-31398 and/or p53 V2 vaccine for a 90 d treatment period. In addition, groups of mice were treated for two sequential 45 d periods with CP-31398 and then V2 vaccine or the reverse for a total treatment period of 90 d (Supplementary Figure S2).

### Statistical Methods

The two-tailed Student's *t* test was performed to interpret the differences between experimental and control groups. Kaplan-Meier and Mantel-Cox Log-Rank analysis was used to calculate significance of survival between untreated control and treated groups of mice.

## SUPPLEMENTARY FIGURES


