# Supplementary material for: Targeting cancer stem cells with p53 modulators
Source: Oncotarget. 2016 Apr 8;7(29):45079–93. doi: 10.18632/oncotarget.8650 (PMC5216707; doi:10.18632/oncotarget.8650)
Supplement: Supplementary file 1 [file oncotarget-07-45079-s001.pdf]

Targeting cancer stem cells with p53 modulators

SUPPLEMENTARY FIGURES

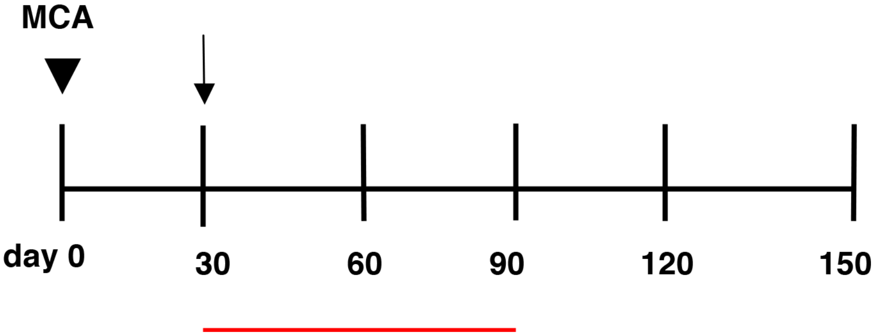

Supplementary Figure S1: Protocol No.1 Combinatorial intervention. Arrow-head indicates the time of MCA challenge of the mice. Arrow indicates the starting time of the administration of the combinatorial intervention. Bold Red line indicates the 60d time course of the intervention. 60 MCA-treated B6/J mice were randomized 30d after challenge into 6 groups of 10 mice each and interventions were initiated and continued for 60d, as indicated above. Group 7 consisted of 10 naïve mice, which received the vaccine for use as controls for monitoring p53 peptide-specific T cells. The experiment was terminated when all MCA mice had tumors that exceeded allowed tumor size limits, necrotic tumors and/or showed decreased health status mandating their removal from a study.

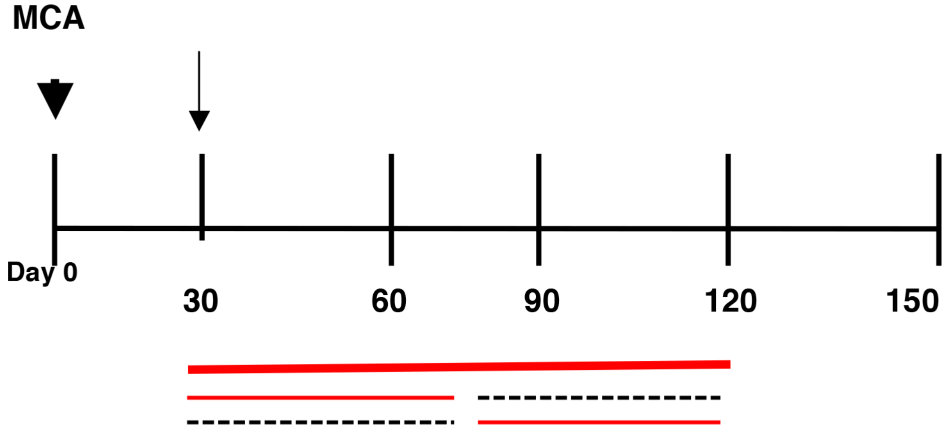

Supplementary Figure S2: Protocol No. 2 Combinatorial intervention: continuous vs. sequential. Bold arrow-head indicates the time of MCA challenge of the mice. Arrow indicates the starting time of administering treatment to the mice. Bold Solid Red ( ) line indicates the 90d time course of the continuous intervention with CP-31398 and p53 V2 vaccine. The dotted black line (-----) indicates treatment with CP-31398 only and solid red line ( ) indicates treatment with p53 V2 vaccine only. The experimental protocol consisted of 6 groups of 10 mice each with therapy initiated 30d post MCA challenge and either continuous intervention for 90 days or 2 segments of 45d each, as indicated above. Group 7 consisted of 10 naïve mice, which received the vaccine for use as controls for monitoring anti-wt p53 peptide-specific T cells. The experiment was terminated when all MCA mice had tumors that exceeded allowed tumor size limits, necrotic tumors and/or showed decreased health status mandating their removal from a study.
